# Supplementary material for: Mitochondrial Respiration of Platelets: Comparison of Isolation Methods
Source: Biomedicines. 2021 Dec 8;9(12):1859. doi: 10.3390/biomedicines9121859 (PMC8698846; doi:10.3390/biomedicines9121859)
Supplement: Supplementary file 1 [file biomedicines-09-01859-s001.zip › biomedicines-1442599-supplementary.pdf]

Table S1. Patient's questionnaire: medical and professional history, physical activity, tobacco and alcohol use. M,male;F,female.

No.	Sex	Age (years)	Body mass (kg)	Height (cm)	Smoking (cigarettes/day)	Alcohol (at least 1 beer/day)	Blood transfusion recipient	Night shifts	Diet	Diabetes	Medicines	Medications for depression/ anxiety	Sport (hours/week)
1	M	35	82	180	1 (2-3)	0	0	0	0	0	0	0	4
2	M	42	88	175	0	0	0	0	0	0	0	0	0
3	M	41	70	170	1 (20)	0	0	0	0	0	Euthyrox	0	0
4	F	28	100	164	1 (5)	0	0	0	0	0	0	0	0
5	M	44	92	183	0	0	0	0	0	0	Prestance 5/5	0	6
6	F	48	63	165	1 (15)	0	1	1	0	0	0	0	7
7	M	40	105	185	0	0	0	0	0	0	0	0	0
8	M	19	97	175	0	1	0	0	0	0	0	0	0
9	M	32	97	188	0	0	0	0	0	0	0	0	0
10	M		90	185	1	0	1	0	0	0	0	0	0
11	M	45	84	178	0	0	0	0	0	0	0	0	3
12	M	46	112	188	0	0	0	1	0	0	0	0	7
13	M	38	84	183	1 (10)	0	0	1	0	0	0	0	0
14	M	39	95	188	0	0	0	0	0	0	Lamiprol	0	8
15	M				0	0	0	1	0	0	0	0	5
16	M	44	89	183	0	0	0	1	0	0	0	0	0
17	M	43	92	185	0	1	0	1	0	0	0	0	0
18	M	31	84	182	0	0	0	0	0	0	0	0	6
19	M	43	95	185	0	0	0	0	0	0	0	0	10
20	M	31	97	186	0	1	0	0	0	0	Desloratadine actavis	0	7
21	M	44	83	174	0	1	0	1	0	0	0	0	0
22	M				1	0	0	0	0	0	Hipres	0	2
23	M	31	100	181	1 (5)	0	0	1	0	0	0	0	2
24	M	42	94	178	0	0	0	0	0	0	0	0	0
25	M	43	93	182	0	0	0	0	0	0	1	0	0

26	M	42	103	198	0	0	0	0	0	0	0	0	0
27	M				0	0	0	0	0	0	0	0	2
28	M	39	84	181	0	0	0	0	0	0	0	0	10
29	M	38	100	187	0	0	0	0	0	0	0	0	8

Table S2. Cell count in samples from different isolation procedures and yield in density gradient (DC) samples. PLT, platelets; PA, platelets apheresis; WA, washed apheresis.

No.	Sex	Number of PLT in whole blood (EDTA) [10^9 x/mL]	Number of PLT in DC sample [10^9 x/mL]	Number of PLT in PA sample [10^9 x/mL]	Number of PLT in WA sample [10^9 x/mL]	Total PLT yield from whole blood using DC [%]
1	M	297	439	128		66.52
2	M	366	534	156		65.66
3	M	342	442	104		58.16
4	F	299	467	97		70.28
5	M	307	451	117		66.11
6	F	266	385	80		65.13
7	M	289	407	113		63.37
8	M	193	345	111		80.44
9	M	311	476	128		68.87
10	M	301	492	118		73.55
11	M	319	424	116		59.81
12	M	320	518	154		72.84
13	M	333	471	144	473	63.65
14	M	297	443	136	587	67.12
15	M	381	666	148	613	78.66
16	M	327	413	137	575	56.83
17	M	279	452	140	658	72.90
18	M	379	574	146	655	68.15
19	M	301	449	145	497	67.13
20	M	240	427	133	550	80.06
21	M	356	526	153	712	66.49
22	M	313	526	128	595	75.62
23	M	275	402	153	606	65.78
24	M	324	459	131	675	63.75
25	M	404	569	145	795	63.38
26	M	358	516	141	677	64.86
27	M	302	421	129	744	62.73
28	M	289	314	115	373	48.89
29	M	187	399	124	597	96.01

Table S3. Statistical comparison of samples from Figure 7. 2-way ANOVA using multiple comparisons of PLT activation markers between PLT isolation methods. The table shows the pairwise comparison with its corresponding *p* values.

Comparison	*p*	Comparison	*p*
**Figure 7A**		**Figure 7B**	
**EDTA (Ctrl) vs. Citrate (Ctrl)**	**0.002**	**EDTA (Ctrl) vs. Citrate (Ctrl)**	**0.029**
EDTA (Ctrl) vs. DC	1	EDTA (Ctrl) vs. DC	0.92
**EDTA (Ctrl) vs. CFA**	**<0.001**	**EDTA (Ctrl) vs. CFA**	**0.012**
EDTA (Ctrl) vs. WA	0.98	EDTA (Ctrl) vs. WA	0.089
**Citrate (Ctrl) vs. DC**	**0.002**	Citrate (Ctrl) vs. DC	0.13
**Citrate (Ctrl) vs. CFA**	**0.029**	**Citrate (Ctrl) vs. CFA**	**0.008**
**Citrate (Ctrl) vs. WA**	**0.001**	Citrate (Ctrl) vs. WA	0.056
**DC vs. CFA**	**<0.001**	**DC vs. CFA**	**0.033**
DC vs. WA	0.97	DC vs. WA	0.19
**CFA vs. WA**	**<0.001**	**CFA vs. WA**	**<0.001**

Figure 7C	
**EDTA (Ctrl) vs. Citrate (Ctrl)**	**0.032**
EDTA (Ctrl) vs. DC	0.24
EDTA (Ctrl) vs. CFA	0.76
EDTA (Ctrl) vs. WA	0.82
**Citrate (Ctrl) vs. DC**	**0.002**
Citrate (Ctrl) vs. CFA	0.22
**Citrate (Ctrl) vs. WA**	**0.016**
**DC vs. CFA**	**0.022**
DC vs. WA	1
CFA vs. WA	0.32

Figure 7D	
**EDTA (Ctrl) vs. Citrate (Ctrl)**	**0.039**
EDTA (Ctrl) vs. DC	1
**EDTA (Ctrl) vs. CFA**	**0.037**
EDTA (Ctrl) vs. WA	0.11
Citrate (Ctrl) vs. DC	0.16
Citrate (Ctrl) vs. CFA	0.92
Citrate (Ctrl) vs. WA	0.34
DC vs. CFA	0.063
DC vs. WA	0.15
CFA vs. WA	0.14

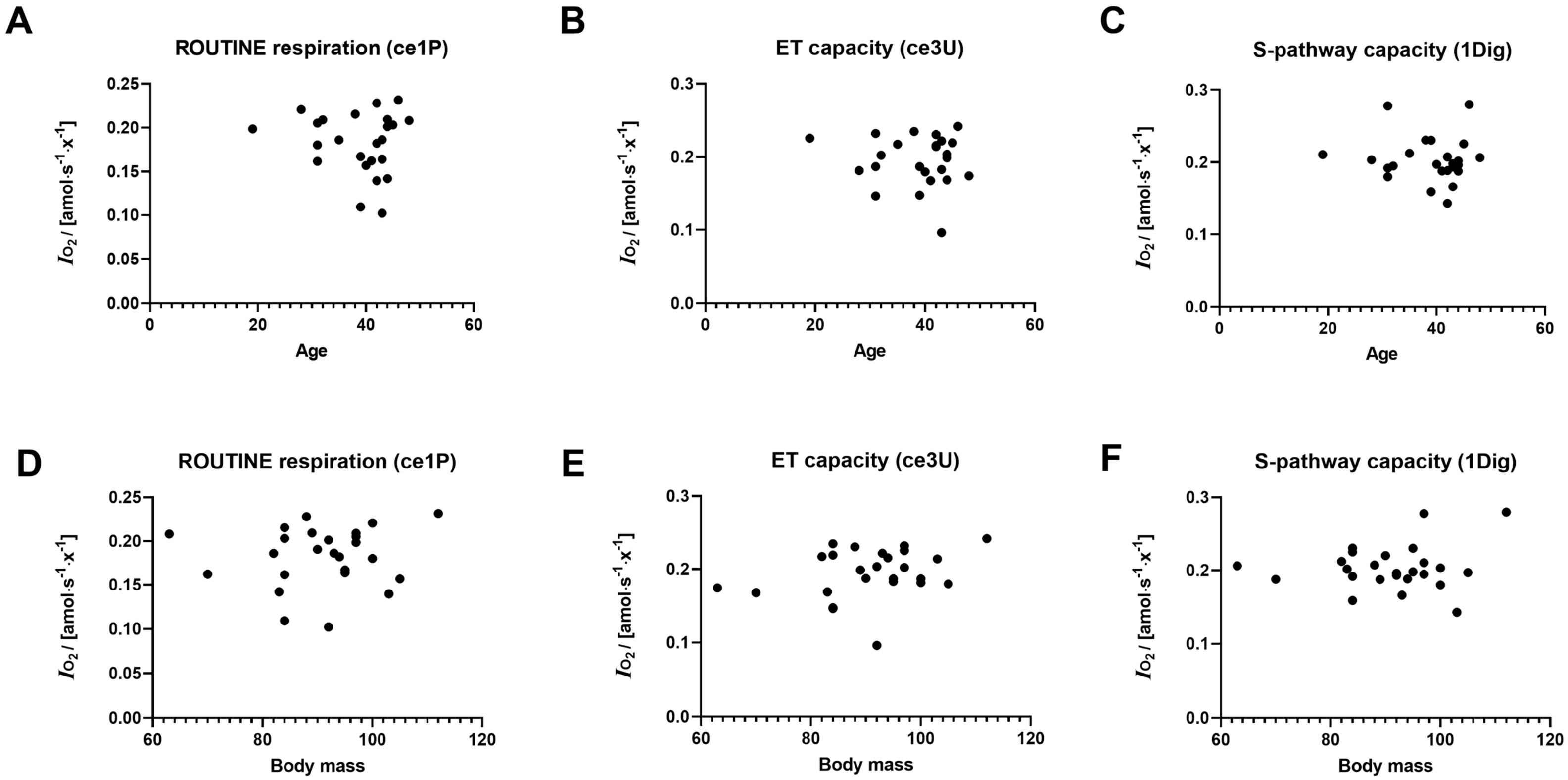

Figure S1. PLT respiration as a function of age and body mass of healty donors. ROUTINE respiration (ce1P), ET capacity of living cells (ce3U), and S-pathway capacity (1Dig) measured in PLT isolated by the DC method. Correlations were not significant with age and body mass.

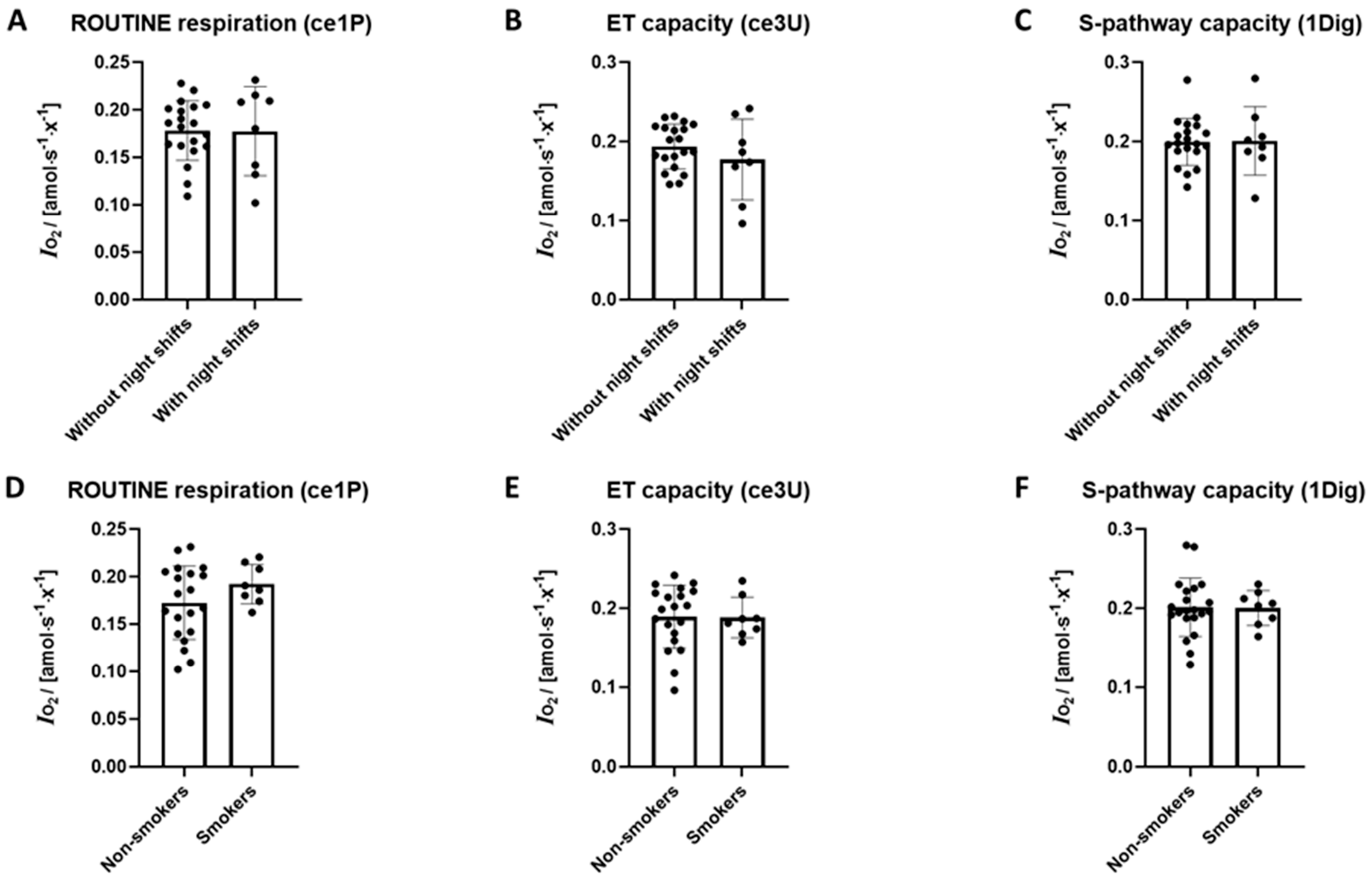

Figure S2. PLT respiration and night shifts or smoking of healthy donors. ROUTINE respiration (ce1P), ET capacity of living cells (ce3U), and S-pathway capacity (1Dig) measured in PLT isolated by the DC method. Difference between groups were not significant.
